# Transcriptome and proteome profiling of neural stem cells from the human subventricular zone in Parkinson’s disease

**DOI:** 10.1186/s40478-019-0736-0

**Published:** 2019-06-03

**Authors:** Vanessa Donega, Saskia M. Burm, Miriam E. van Strien, Emma J. van Bodegraven, Iryna Paliukhovich, Hanneke Geut, Wilma D. J. van de Berg, Ka Wan Li, August B. Smit, Onur Basak, Elly M. Hol

**Affiliations:** 1 Department of Translational Neuroscience, UMC Utrecht Brain Center, University Medical Center Utrecht, Utrecht University, Utrecht, The Netherlands; 2 Department of Molecular and Cellular Neurobiology, Center for Neurogenomics and Cognitive Research, Neuroscience Campus Amsterdam, VU University, Amsterdam, The Netherlands; 3 Department of Anatomy and Neurosciences, Amsterdam Neuroscience, Amsterdam UMC, VU University Medical Center, Amsterdam, The Netherlands; 4 Hubrecht Institute, Royal Netherlands Academy of Arts and Sciences and University Medical Center Utrecht, Utrecht, The Netherlands; 5 Department of Neuroimmunology, Netherlands Institute for Neuroscience, Institute of the Royal Netherlands Academy of Arts and Sciences, Amsterdam, The Netherlands

**Keywords:** RNA sequencing, Transcriptomics, Proteomics, Neural stem cells, Quiescence, Human SVZ, Parkinson’s disease

## Abstract

It is currently accepted that the human brain has a limited neurogenic capacity and an impaired regenerative potential. We have previously shown the existence of CD271-expressing neural stem cells (NSCs) in the subventricular zone (SVZ) of Parkinson’s disease (PD) patients, which proliferate and differentiate towards neurons and glial cells in vitro. To study the molecular profile of these NSCs in detail, we performed RNA sequencing and mass spectrometry on CD271^+^ NSCs isolated from human post-mortem SVZ and on homogenates of the SVZ. CD271^+^ cells were isolated through magnetic cell separation (MACS). We first compared the molecular profile of CD271^+^ NSCs to the SVZ homogenate from control donors and then compared CD271^+^ cells to CD11b^+^ microglia. These results confirmed their neural stem cell identity. Finally we compared controls and PD patients to establish a specific molecular profile of NSCs and the SVZ in PD. While our transcriptome analysis did not identify any differentially expressed genes in the SVZ between control and PD patients, our proteome analysis revealed several proteins that were differentially expressed in PD. Some of these proteins are involved in cytoskeletal organization and mitochondrial function. Transcriptome and proteome analyses of NSCs from PD revealed changes in the expression of genes and proteins involved in metabolism, transcriptional activity and cytoskeletal organization. Our data suggest that NSCs may transit into a primed-quiescent state, that is in an “alert” non-proliferative phase in PD. Our results not only confirm pathological hallmarks of PD (e.g. impaired mitochondrial function), but also show that the NSCs from SVZ undergo significant changes at both transcriptome and proteome level following PD.

**Electronic supplementary material:**

The online version of this article (10.1186/s40478-019-0736-0) contains supplementary material, which is available to authorized users.

## Introduction

Parkinson’s disease (PD) is characterized by progressive degeneration of dopaminergic neurons resulting in significant impairments of both cognitive and motor function [51]. Current therapeutic options, such as L-Dopa treatment, triggers side-effects with prolonged use of the drug [7]. It is therefore, crucial that new therapeutic options are developed, which lead to long-term improvement of both motor and cognitive functions. An alternative treatment option would be the replacement of lost dopaminergic neurons by stimulating endogenous neural stem cells (NSCs) to generate new dopaminergic neurons. The challenge for this alternative is that the mammalian brain has an impaired capacity to repair. Following birth, neurogenesis in humans becomes restricted to two germinally active regions: the subventricular zone (SVZ) along the lateral ventricles and the subgranular zone (SGZ) of the hippocampal dentate gyrus [13, 14, 23]. Importantly, we found evidence that a population of NSCs persist in the SVZ of both elderly [30, 52] and PD patients [54]. Our previous studies show that these NSCs, which are GFAPδ and NGFR (i.e. CD271) positive, proliferate and differentiate towards neurons and glial cells in vitro [44, 52]. These findings offer hope for the development of therapeutic strategies to stimulate these NSCs to regenerate the injured brain.

Studies in rodents suggest that activation of endogenous NSCs leads to the replacement of lost dopaminergic neurons [5, 27–29]. Endogenous NSCs may thus be a source of stem cells for brain repair to improve cognitive function. Developing an approach to stimulate the endogenous capacity of the brain to repair also has advantages, as it circumvents the risks and ethical concerns of cell-replacement based strategies in which fetal or induced pluripotent stem cell-derived NSCs are transplanted into the injured brain [3, 32, 53].

Due to the scarcity of human brain material and technical limitations, it remains unresolved whether repair occurs in the human brain. Despite the lack of conclusive evidence for neurogenesis following injury in humans [36], the fact that NSCs can be found in germinal regions in the human brain raise new possibilities for the development of therapeutic strategies [23, 46, 47, 50]. However, little is known about the nature of adult human NSCs due to the limitations mentioned above. Recent advances in transcriptome analysis offer an unprecedented level of resolution into the molecular signature of specific cell types. Recently, single cell RNA sequencing has provided novel insights into the molecular profile and behavior of radial glial cells (RGCs) from the rodent [12, 34, 49, 57] and fetal human SVZ [22, 58]. These studies revealed that RGCs are a highly heterogeneous population of cells.

In this study, we isolated CD271^+^ NSCs from human post-mortem SVZ tissue from control and PD donors, [55] and assessed their molecular profile by RNA sequencing and mass spectrometry. First, we compared the molecular profile of CD271^+^ NSCs to the SVZ homogenate and CD11b^+^ microglia to determine their gene signature profile. We next compared the transcriptome of CD271^+^ cells to CD11b^+^ microglia, which confirmed the NSC identity of the CD271^+^ cells and provide a molecular signature of NSCs of the adult human SVZ. The expression profiles of CD271^+^ cells and SVZ homogenate were compared between controls and PD patients to establish a molecular profile of NSCs and the SVZ following PD. Transcriptome and proteome analysis revealed changes in the expression of genes and proteins involved in metabolism, cytoskeletal organization and transcriptional activity.

## Material and methods

### Human post-mortem brain tissue

Human post-mortem SVZ tissue from PD patients (*n* = 16) and donors without neurological disease (i.e. control donors) (*n* = 11) were obtained from the Netherlands Brain Bank (NBB; https://www.brainbank.nl). The NBB is specialized in performing quick brain autopsies (average post-mortem delay of samples included in this study is 7 h), which is a prerequisite to ensure excellent tissue quality. NBB has permission from the donors to perform autopsies for tissue isolation and accessing medical records for scientific purposes. All donors, or their next of kin, have given informed consent for the use of brain tissue for scientific purposes. To ensure donor anonymity we only disclose a serial number, which is given by the NBB. This number relates to the year the autopsy was performed and the autopsy number in that year. We obtained freshly dissected SVZ tissue (1–5 g per donor) of Parkinson’s patients (PD) and control (Cntr) donors. Clinico-pathological information of all donors was gathered (Additional file 1: Table S1, Additional files 8 and 9: Figure S1 and Figure S2). The clinical diagnosis was confirmed by post-mortem pathological evaluation of several brain regions, which included evaluation of Alzheimer’s and PD pathology according to the Braak staging system, Brain Net Europe protocols and ARTAG [1, 39]. For the RNA sequencing analysis there was a significant difference in the mean age of the donors (Cntr = 84 years; PD = 76 years) (Additional file 8: Figure S1) and in the distribution of males and females in the groups (Additional file 8: Figure S1 and S2). Control donors were only included to the analysis, following assessment of medical history and pathological scoring. Because these are all aged donors, it is practically impossible to have control donors with a score of 0 on Thal_phase and Braak NFT. Donors used for RNA sequencing showed no significant difference in Braak NFT and CERAD scores, but both Thal_phase and Braak α-syn scores were significantly higher in the PD donors (Additional file 8: Figure S1). Donors used for proteomics analysis only differed significantly in Braak α-syn scores (Additional file 9: Figure S2). Following pathological evaluation by the NBB, it appeared that one control donor that was used for RNA sequencing of the SVZ had early signs of PD pathology in the brainstem, as shown on the Braak α-syn and McKeith α-syn scores. As this was not associated with cognitive and motor impairments we kept this donor as a control.

### Immunohistochemistry and quantification

Immunohistochemistry was performed on post-mortem SVZ tissue (Additional file 2: Table S2) post-fixed in 4% paraformaldehyde (PFA) dissolved in PBS. Briefly, 7 μm thin paraffin sections were deparaffinated, washed, and antigen retrieval was performed in 10 mM citrate buffer pH 6.0 at 80 °C for 20 minutes (min). After cooling down, sections were thoroughly washed in 0.25% Triton-X in PBS and incubated with blocking solution 0.25% BSA with 0.4% Triton-X in PBS at room temperature for 1 hour (h). Sections were incubated with primary antibodies at 4 °C overnight. The following primary antibodies were used: rabbit anti-FGFR3 (1:100; Santa Cruz sc-9007) and rabbit anti-S100β (1:1000; Swant). The next day after thorough washing, the sections were incubated with the corresponding secondary antibodies conjugated to Alexa-555 (1:1000, Invitrogen) at room temperature for 1 h. Subsequently, the sections were incubated with Sudan Black to quench autofluorescence for 7 min and washed in 70% ethanol for 1 min. Nuclear counterstaining was done with Hoechst 33258 (1:1000, Biorad). The stained sections were analyzed on a Zeiss LSM880 confocal laser microscope using 40x/ 1.3NA oil DICII objectives (EC PlnN), a AxioCam MRm camera (Zeiss), and the software Zen black Z.1SP3. Images were taken with a z-step of 2 μm and a resolution of 1024 × 1024. FGFR3 and S100β positive cells in the SVZ were counted by eye in one section of the SVZ and corrected for the area (SVZ area ranged from 2 to 7 mm^2^).

### Cell isolation

CD271^+^ NSCs and CD11b^+^ microglia were isolated from post-mortem human SVZ tissue as described previously [55]. Briefly, all visible blood vessels were removed and the tissue was dissociated mechanically and enzymatically using 2.5% trypsin (Gibco, Life Technologies, Paisley, UK) and 20 U/ml DNase (Roche Diagnostics GmbH, Mannheim, Germany) at 37 °C for 30 min. The tissue homogenate was washed and resuspended in cold DMEM without phenol red (Gibco, Life Technologies), and filtered through a 100 μM nylon cell strainer (Corning, New York, USA). Next, Percoll (GE Healthcare Bio-sciences AB, Uppsala, Sweden) density gradient centrifugation (30 min at 3200 x g at 4 °C) was used to separate the different cellular fractions. The turbid fraction containing NSCs (second fraction with lowest density) was collected and washed in complete DMEM (Gibco, Life Technologies) supplemented with 10% fetal calf serum (FCS), 25 mM HEPES pH 7.2, 25 μg/ml penicillin, 25 μg/ml streptomycin. CD271^+^ NSCs and CD11b^+^ microglia were isolated with magnetic cell separation (MACS) (Miltenyi Biotec, Bergisch Gladbach, Germany) by using microbeads coated with an antibody against CD271 or CD11b (Miltenyi Biotec). This procedure yielded between 25.000–100.000 CD271^+^ NSCs and 500.000–1.000.000 CD11b^+^ microglia per donor. SVZ samples were generated on unsorted SVZ tissue by homogenization in Trizol (Ambion, Life Technologies, Carlsbad, CA). Following centrifugation, the cell pellets were either lysed in Trizol at room temperature for 15 min and stored at − 80 °C until RNA sequencing analysis or stored directly at − 80 °C for mass spectrometry analysis.

### RNA sequencing

#### RNA isolation and quality control

Total cellular RNA was isolated using the miRNeasy mini kit in combination with DNase treatment (Qiagen GmbH, Hilden, Germany) according to the manufacturers’ instructions. Total RNA was eluted in 10 μl RNase-free water and RNA concentration was measured on a Nanodrop (Thermo Scientific). The RNA Integrity Number (RIN) value was determined using the Agilent RNA 6000 p kit (Agilent Technologies, Waldbronn, Germany) on the Agilent 2100 Bioanalyzer according to the manufacturers’ protocol. When possible only samples with a RIN value higher than 6 were included (Additional file 3: Table S3). We did not observe any effect of lower RIN value on total number of reads after mapping following RNA sequencing (Additional file 3: Table S3).

#### Cel-Seq2 library preparation and sequencing

For each sample a custom-made primer solution was added to 15 ng RNA, which was subsequently denatured at 70 °C for 2 min, and quickly cooled thereafter. mRNA was reverse transcribed into cDNA (first and second strand) using the Cel-Seq kit and purified using clean up columns (ThermoFisher Scientific, Ambion, Waltham, USA). The purified cDNA was transcribed in vitro to obtain amplified RNA, which was followed by purification with Agencourt RNAClean XP (RNAse free) beads (Beckman Coulter) and ExoSap treatment to remove residual primers (Thermo Fisher Scientific). aRNA was fragmented using the Cel-Seq kit (Ambion) and purified using RNAClean XP (RNAse free) beads. Quality of the amplified RNA was assessed by Agilent 2100 Bioanalyzer measurements using RNA pico chips (Agilent). Next, cDNA libraries were made from the amplified RNA using a modified Cel-Seq2 method [18]. The amplified RNA was reverse transcribed and amplified by PCR. Libraries were labeled with a 4 bp random unique molecular identifier (UMI) that was added to the primer in between the cell-specific barcode and the poly-T stretch. Libraries were purified twice using AMPure XP Beads (Beckman Coulter). Quality of the DNA libraries was assessed by Bioanalyzer measurements using high sensitivity DNA chips (Agilent). Libraries were sequenced on Illumina Nextseq 500 and Illumina HighSeq 2500 using paired-end sequencing.

#### Data analysis

Paired-end reads from Illumina sequencing were aligned to the human genome with Burrows-Wheeler Aligner (BWA) [31] (version 0.7.10-r126) and assigned to the correct libraries. Reads that mapped equally well to multiple loci were discarded. For each sample, all transcripts derived from the same gene locus were aggregated. Libraries for RNA sequencing were prepared by matching samples that were comparable in RIN profile and RNA concentration (Additional file 3: Table S3). There was no correlation between read count and age of the donors, disease status, post-mortem delay, cell type, or RIN values of the RNA (not shown). Upon alignment of the raw RNAseq reads, we excluded samples that had fewer than 500.000 reads (Additional file 3: Table S3). Read counts were normalized by using transcripts per million (TPM). A total of 21 samples, including six CD11b^+^ (2 Control and 4 PD), eight CD271^+^ samples (3 Control and 5 PD) and seven SVZ samples (3 Control and 4 PD) were analyzed. Differentially expressed genes were identified with R studio package DESeq2.

### Gene ontology enrichment analysis

The gene ontology (GO) analysis, Panther pathway analysis, and Protein Protein Interaction (PPI) analysis were performed on a list of differentially expressed genes that was obtained by performing differential gene expression analysis with R studio package DESeq2 (both down- or up-regulated genes). The lists of genes used for this analysis are provided as Additional files 5, 6 and 7: Tables S5-S7 and provide the list of differentially expressed genes in CD271^+^ NSCs vs SVZ from control donors, CD271^+^ NSCs vs CD11b^+^ microglia from both control and PD donors and in CD271^+^ NSCs from control vs PD donors. We loaded these lists in EnrichR web-based tool [6, 26] (http://amp.pharm.mssm.edu/Enrichr/), which performs enrichment analysis. The selection of NSC, oligodendrocyte and neuroblast genes for the heatmaps was based on a single cell RNA sequencing dataset that identified these population of cells in the mouse brain [34] using the Morpheus Broad Institute Software (https://software.broadinstitute.org/morpheus/).

### qRT-PCR

From the residual RNA, cDNA was synthetized using the Quantitect Reverse Transcription kit (Qiagen GmbH, Hilden, Germany) according to manufacturers’ protocol. Primers were designed using the Primer-BLAST designing tool from the NCBI website (https://www.ncbi.nlm.nih.gov/tools/primer-blast/; listed in Additional file 4: Table S4) and qRT-PCRs were performed on the Quantistudio 6 Flex (Applied Biosystems, Life Technologies) using FastStart Universal SYBR green master (Rox) reagent (Roche Diagnostics) and analyzed using the Quantstudio Realtime PCR software (version v1.1; Applied Biosystems). Relative gene expression was normalized to the reference genes *E2Ubi*, *Alus,* and *Ef1a* using the Pfaffl method [42].

### Mass spectrometry

#### Sample preparation

The pellets of NSCs (Control *n* = 6; PD *n* = 7) and fresh frozen whole SVZ (Control *n* = 3; PD n = 7) were homogenized in 2x sodium dodecyl sulfate (SDS) buffer and heated to 98 °C for 5 min, and 3 μL 30% acrylamide was added to alkylate the cysteine. Samples were loaded onto a 10% SDS-polyacrylamide gel and run briefly for 10 min. Gels were stained lightly with colloidal Coomassie Blue, and the gel lane of each sample was cut out and chopped into smaller pieces using a scalpel and transferred to an Eppendorf tube. The gel pieces were destained with 50% acetonitrile in 50 mM ammonium bicarbonate, dehydrated in 100% acetonitrile, and rehydrated in 50 mM ammonium bicarbonate. This destaining cycle was repeated once. After dehydration in 100% acetonitrile, the samples were incubated with trypsin solution containing 10 μg/ml trypsin (sequence grade; Promega, Madison, WI, USA) in 50 mM ammonium bicarbonate at 37 °C overnight. Peptides from the gel pieces were extracted twice with 100 μl 50% acetonitrile in 0.1% trifluoroacetic acid, dried in a speedvac and stored at − 20 °C until further use.

#### Mass spectrometry

Peptides were redissolved in 7.5 μL 0.1% formic acid and analyzed by mass spectrometry according to protocols previously described [41] using the TripleTOF 5600 mass spectrometer (Sciex, Ontario, Canada) coupled to an Ultimate 3000 LC system (Dionex, ThermoFisher Scientific). Peptides were fractionated on a 200 mm Alltima C18 column (300 μm ID, 3 μm particle size) at a flow rate of 5 μl/min. Acetonitrile concentration in the mobile phase in 0.1% formic acid was increased from 5 to 18% in 88 min, to 25% at 98 min, 40% at 108 min, and to 90% at 110 min. The MS survey scan range was m/z 350–1250, with top 20 precursor ions selected for MS/MS acquisition. Rolling CID function was activated. All raw MS data were analyzed by MaxQuant software (version 1.5.2.8) with search engine Andromeda. The Human database used was UniProt_2015–02. The fixed modification was propioamide. Match between runs with match time window of 0.7 min and alignment time window of 5 min were used for all analyses. For other parameters the default settings were used.

#### Data analysis

The mass spectra were searched against the Swissprot human database (version Feb 2015) with MaxQuant software (version 1.6.2.3). The mass tolerances in MS and MS/MS were set to 6 ppm and 0.5 Da, respectively. Trypsin was selected as the digestion enzyme and up to two missed cleavages were allowed. Methionine oxidation and protein N-terminal acetylation were set as variable modifications, and cysteine alkylation with acrylamide was set as fixed modification. False discovery rates of both peptides and proteins were set within a threshold value of 0.01. The valid protein hits should contain at least one unique peptide. The intensity-based absolute quantification (iBAQ) was calculated by dividing the summed MS intensity of all assigned peptides for each protein by the number of theoretically observable peptides. Only unique peptides were used for identification and quantitation. *P*-values and logFC were calculated with the R studio package Limma (version 3.36.1).

### Statistical analysis

Data are expressed as mean ± SEM. Significance was tested on GraphPad Prism 7 with two-tailed unpaired t-test or one-way ANOVA followed by Sidak’s multiple comparisons test.

## Results

### Transcriptome analysis of the human SVZ

**Fig. 1:**
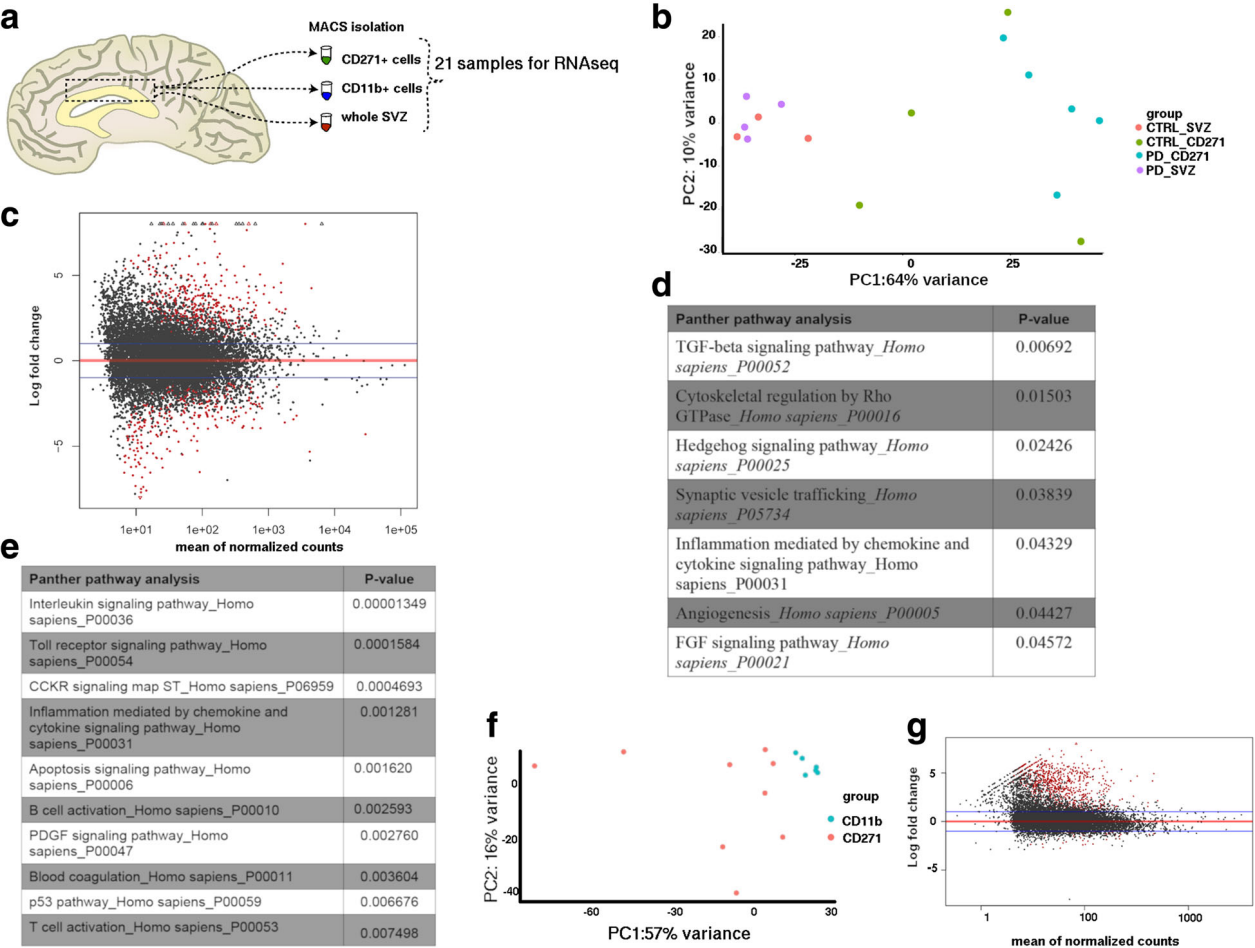
Transcriptome profiling of neural stem cells of the human SVZ. **a** Scheme of experimental set-up. **b** PCA plot showing distribution of SVZ and CD271^+^ NSCs from both control and PD donors. **c** Differentially expressed genes (in red) visualized in an MA plot for CD271^+^ cells vs SVZ cells from control donors (*p*-value < 0.01, adj p-value < 0.01). **d** Panther pathway analysis of differentially expressed genes between CD271^+^ cells and SVZ cells (p-value < 0.05). **e** Panther pathway analysis of genes upregulated in CD271^+^ cells when compared to SVZ homogenate (p-value < 0.05). **f** PCA plot showing the distribution of CD271^+^ cells and CD11b^+^ cells. **g** MA plot showing differentially expressed genes (in red) between CD271^+^ cells and CD11b^+^ cells. CTRL = control; PD = Parkinson’s disease

Transcriptome analysis was performed on sorted CD271^+^ NSCs, CD11b^+^ cells, and total SVZ tissue (from here on called SVZ homogenate) from controls and PD patients (Fig. 1a). We started by comparing the CD271^+^ NSCs with the SVZ homogenate. A principle component analysis (PCA) revealed one well-defined cluster, corresponding to the SVZ homogenate (Fig. 1b). In contrast, no clusters were identified in the CD271^+^ NSCs, suggesting heterogeneity. This heterogeneity is observed in both control and PD patients and is thus, not due to differences in disease progression. Differential gene expression analysis identified 605 genes that were differentially expressed in CD271^+^ NSCs when compared to the SVZ cell population (adj *p*-value < 0.01; Fig. 1c and Additional file 5: Table S5). Of these differentially expressed genes 334 were higher expressed in CD271^+^ cells in comparison to SVZ homogenate, indicating that these are the signature genes of the NSCs.

Panther pathway analysis on genes downregulated in CD271^+^ cells identified enrichment for genes involved in TGF-β, Hedgehog, and FGF signaling pathways, which are important regulators of neurogenesis and cell cycle progression [8] (Fig. 1d). Panther pathway analysis on genes upregulated in CD271^+^ cells showed enrichment for inflammation and apoptosis signaling pathways (Fig. 1e), thus hinting that we isolated an immune cell population. Therefore, we next compared these cells to CD11b^+^ microglia, which confirmed the stem cell identity of CD271^+^ cells (adj *p*-value < 0.01; Fig. 1f and Additional file 6: Table S6). Differential gene expression analysis identified 895 genes that were differentially expressed in CD271^+^ cells when compared to CD11b^+^ microglia (Fig. 1g and Additional file 6: Table S6). 825 of these genes were upregulated in CD271^+^ cells, among which the neural stem cell markers *SOX2* and *NES*. Gene ontology (GO) analysis on these upregulated genes showed enrichment for GO terms related to central nervous system development and glial cell differentiation (not shown). Differentially expressed genes between CD271^+^ cells and SVZ homogenate, further confirmed the phenotype of the isolated populations of cells, with CD271^+^ cells showing the expression of NSC markers, such as *CD9*, *ID2*, *EGFR* and *MCM2*. As expected, the SVZ homogenate showed expression of oligodendrocyte (e.g. *Sox10* and *Olig1*), NSC and neuroblast (e.g. *DCX* and *TUBB3*) markers (Additional file 10: Figure S3).

### Changes in transcript profile of CD271^+^ NSCs following Parkinson’s disease

**Fig. 2:**
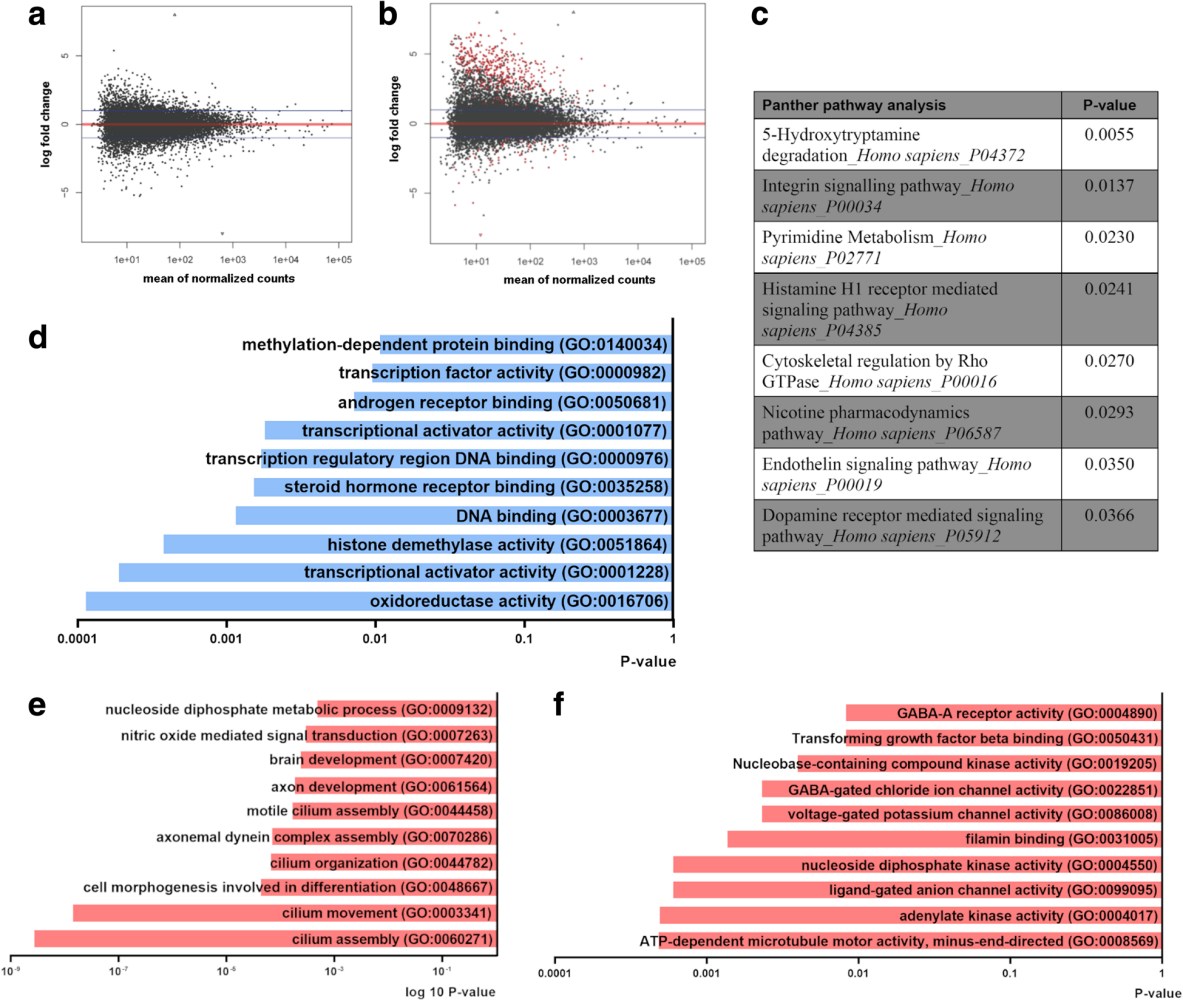
Differential gene expression in CD271^+^ cells following PD. **a-b** MA plots showing no differentially expressed transcripts between SVZ from controls and PD patients (**a**) and CD271^+^ NSCs from controls and PD patients (**b**) (p-value < 0.01; adj p-value < 0.05). **c** Panther pathway analysis highlights changes in serotonin (i.e. 5-hydroxytryptamine) degradation and dopamine signaling (p-value < 0.05). **d** GO analysis of upregulated genes in CD271^+^ NSCs showing changes in oxidoreductase activity and transcriptional activity. **e** GO biological process analysis of downregulated genes highlights cilium movement and assembly. **f** GO molecular function analysis of downregulated transcripts showing dysregulation of channel function, TGF-β binding and cytoskeletal organization. Cntr = control; PD = Parkinson’s disease

Next, we determined the changes in the transcriptome that occurred in PD. No significant differences in gene expression between controls and PD patients were found in the SVZ homogenate (Fig. 2a). In contrast, we identified 483 genes that were differentially expressed in CD271^+^ NSCs from PD patients compared to control donors (Fig. 2b). The expression of 61 genes was significantly increased, while the expression of 422 genes was decreased (Additional file 5: Table S5) in PD patients. A selection of top upregulated and downregulated transcripts (viz. *KDMA2A*, *RAD51C, HSPA1L*, *IGFBP5* adj. *p*-value < 0.005) was validated by qPCR (Additional file 11: Figure S4). These genes encode for proteins involved in protein demethylation, mtDNA integrity, mitochondrial quality control and tissue development [19, 35, 38, 45].

Interestingly, Panther pathway analysis highlighted changes in serotonin degradation and dopamine signaling due to downregulation of *MAOB*, *ALDH3A1, ADCY2, CLIC6* and *PPP1R1B* genes (Fig. 2c). GO analysis of upregulated genes in PD-derived CD271^+^ NSCs revealed changes in the “oxidoreductase activity” and “transcriptional activity” (Fig. 2d). Moreover, GO analysis of downregulated genes showed changes in “TGFβ binding”, “filamin binding” and “cilium assembly and movement” (Fig. 2d-e). Our results underline key changes in the transcriptome signature of CD271^+^ NSCs in PD, among which are cytoskeletal organization, transcriptional activity and channel activity.

**Fig. 3:**
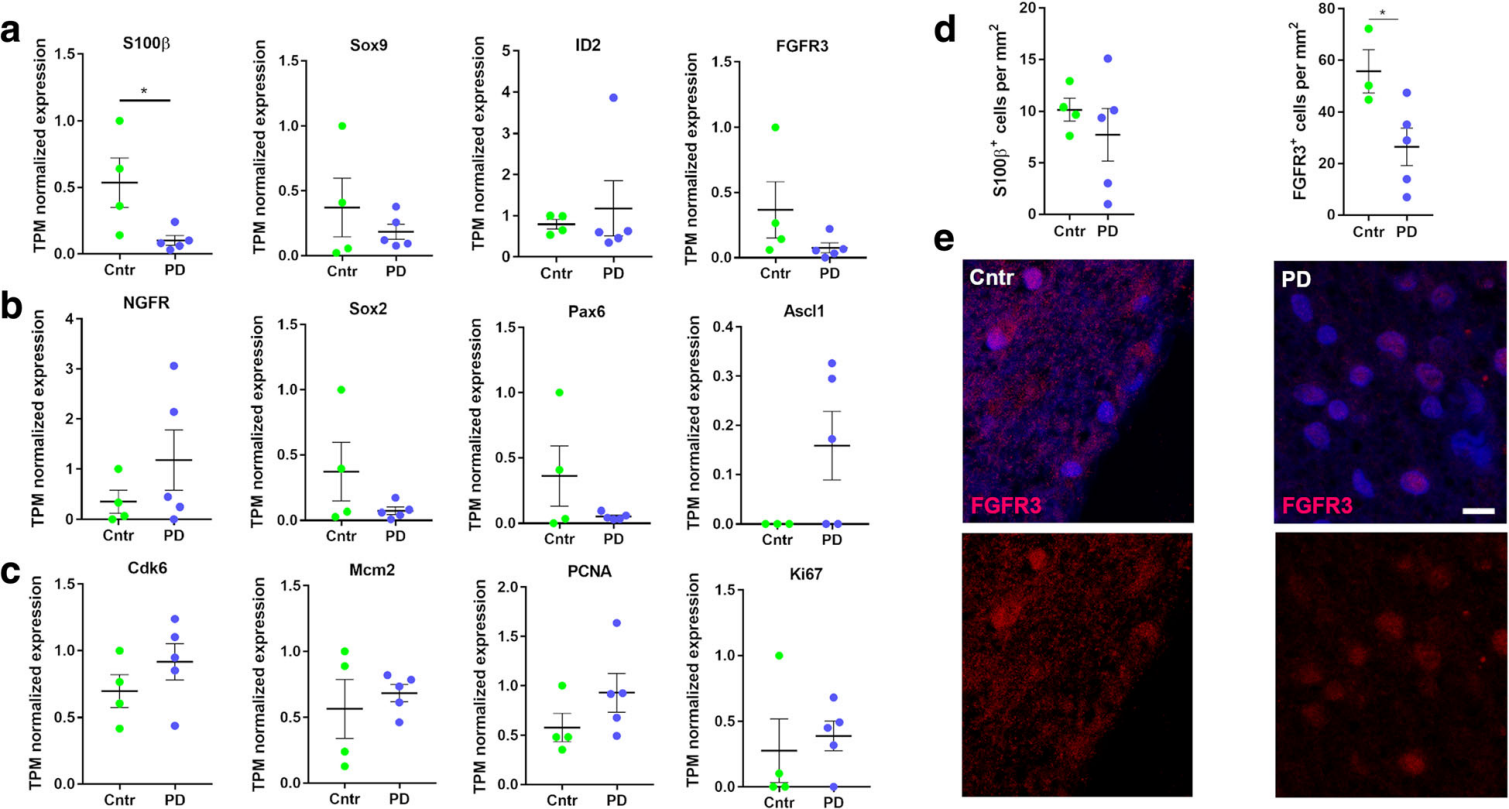
Expression of genes involved in NSC activation state remains unaltered after PD. **a-c** Relative expression of markers for qNSCs and aNSCs (**a**), lineage progression (**b**) and proliferation (**c**). Data shown are TPM-normalized read counts. **d** Quantification of the number of S100β^+^ and FGFR3^+^ cells in the human SVZ (control and PD). **e** Representative images of FGFR3^+^ cells in the human SVZ. Cntr = control; PD = Parkinson’s disease. Data are presented as mean ± SEM. Scale bar = 10 μm

It has been shown in adult rodents, that NSCs are a dynamic population of cells that transit between different activation states, i.e. dormant/quiescent (qNSC), primed-quiescent, and aNSC [9, 16, 34]. Interestingly, qNSCs were shown to re-enter the cell cycle through interferon (IFN)-γ mediated activation following ischemic brain lesion in adult mice [34]. Thus, to determine whether the dysregulated gene expression could be associated with changes in NSC cell cycle transition, i.e. quiescent to activated state, we assessed gene expression of specific markers for qNSCs/astrocytes, lineage specification, and proliferation [12, 48], showed a decrease in the expression of astrocyte marker *S100β* (Fig. 3a). No significant changes were observed for the expression of lineage markers (Fig. 3b). Proliferation markers did not increase significantly (Fig. 3c) as previously shown by immunohistochemical analysis on SVZ tissue sections from PD patients [54]. Immunohistochemical validation showed a significant decrease in the expression of FGFR3, a marker for quiescence in the SVZ of PD patients compared to control donors (Fig. 3d-e). All together, our data suggest that CD271^+^ NSCs may transit into a primed-quiescent state in PD.

### Changes in proteins involved in metabolism in CD271^+^ NSCs in Parkinson’s disease

**Fig. 4:**
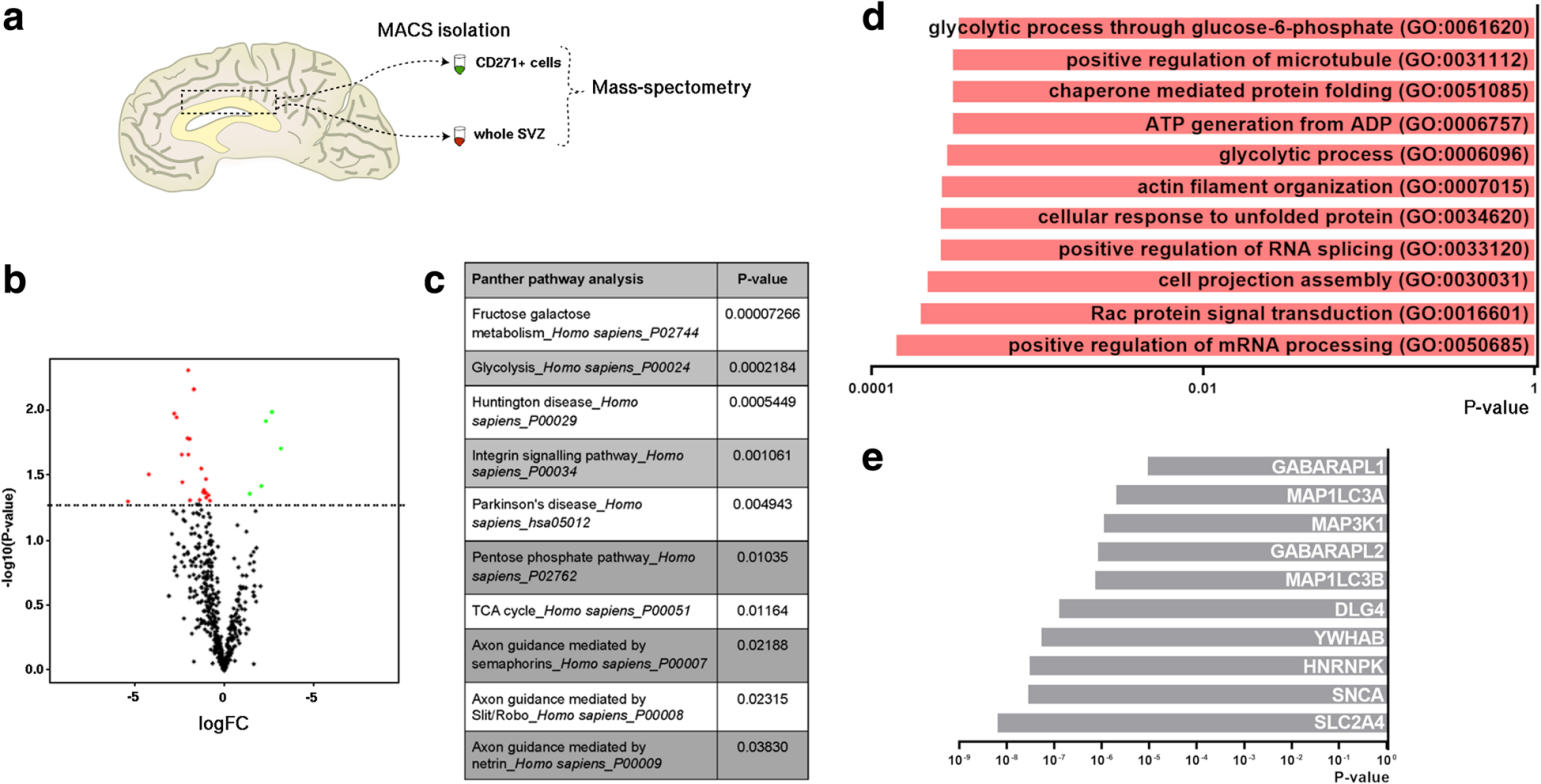
Proteomics analysis reveals differential protein expression in CD271^+^ NSCs of PD patients. **a** Schematic of experimental set-up. **b** Vulcano plot showing differentially expressed proteins in CD271^+^ NSCs of PD patients (red = downregulated; green = upregulated) (p-value < 0.05; logFC > 0.1). **c** Panther pathway analysis showing changes in signaling pathways involved in metabolism and PD (p-value < 0.05). **d** GO analysis (biological process) further highlights dysregulation of metabolic processes. **e** Protein-protein interaction (PPI) analysis showing the top 10 proteins to interact with downregulated proteins

We next assessed whether there were any significant changes in protein expression in CD271^+^ NSCs in PD, by mass spectrometry analysis of CD271^+^ NSCs of control (*n* = 6) and PD patients (*n* = 7) (Fig. 4a). Proteomics analysis included 480 proteins, as we only selected proteins that were detected in at least 5 out of 6 donors. Linear regression analysis identified 26 proteins that were differentially expressed in PD (Fig. 4b and Additional file 7: Table S7). Interestingly, Panther pathway analysis on these proteins showed changes in signaling pathways involved in “metabolism” (ALDOA, HK1, MDH1), and “neurodegenerative diseases” (VAT1, RHOG, RAC1, HSPA9, HSPA1A) (Fig. 4c and Additional file 11: Figure S5). ALDOA and HK1 are both key enzymes in glucose metabolism. HK1 is also significantly downregulated at gene level (Additional file 12: Figure S5a). Interestingly, the protein expression level of some ATP synthases was increased in PD (Additional file 12: Figure S5a). These ATP synthases are involved in producing ATP during oxidative phosphorylation. Changes in metabolic processes were further highlighted by GO analysis (Fig. 4d). To assess which protein networks the downregulated proteins could be part of, protein-protein interaction (PPI) analysis was performed on the EnrichR platform to determine proteins that could potentially interact with the identified downregulated proteins. This analysis showed that proteins involved in “glucose transport” (SLC2A4), “mRNA processing” (HNRNPK) and “cell cycle” (YWHAB) were among the top 10 proteins that could potentially interact with the downregulated proteins (Fig. 4e).

**Fig. 5:**
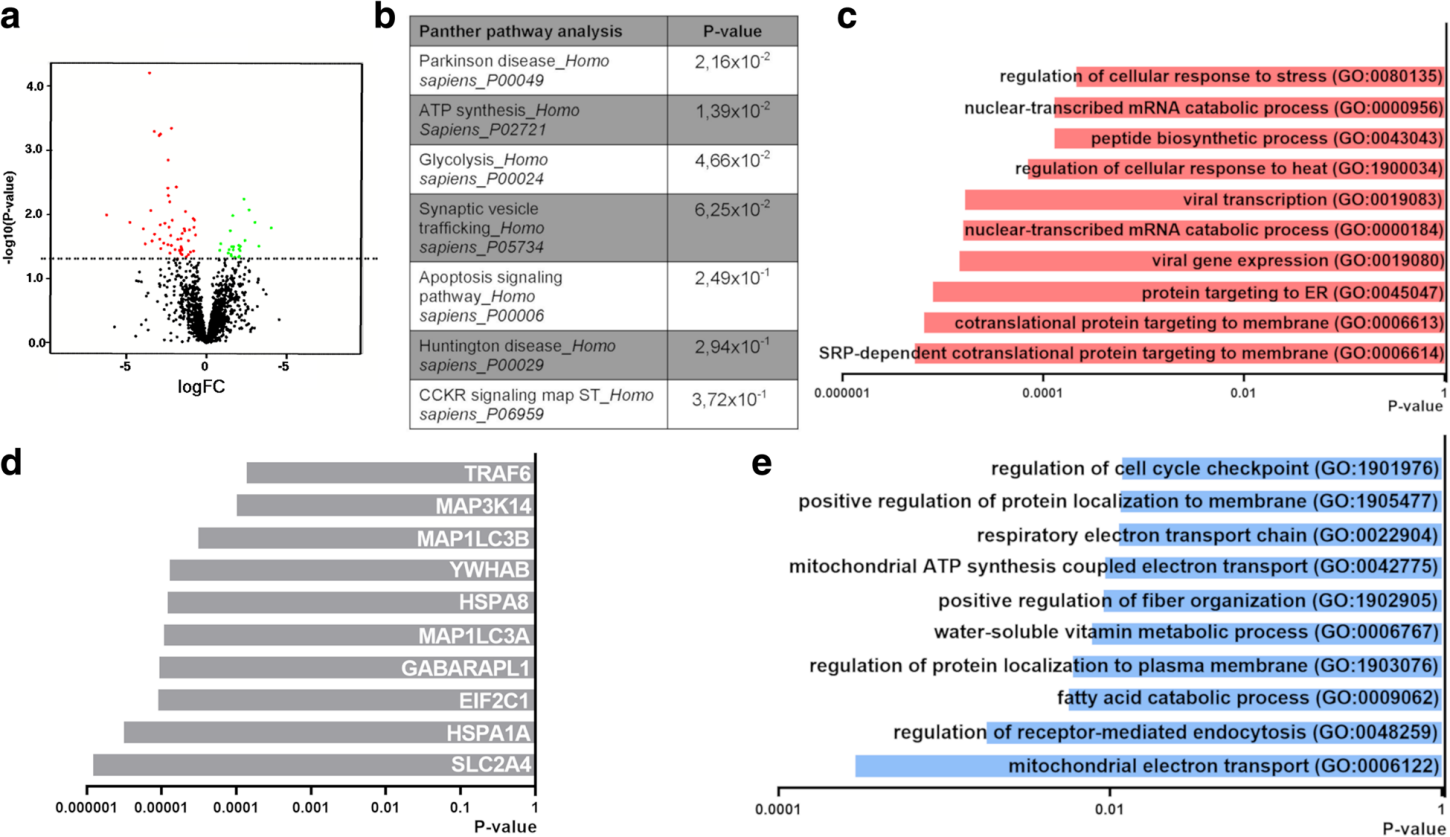
Changes in protein expression in the SVZ of PD patients. **a** Vulcano plot showing differentially expressed proteins in the SVZ of PD patients (*p* < 0.05; logFC > 0.1). **b** Panther pathway analysis showing changes in signaling pathways involved in “Parkinson’s disease”, “glycolysis” and “ATP synthesis” (p-value < 0.5). **c** GO analysis (biological process) of downregulated proteins suggests dysregulation in “protein translocation machinery”. **d** Protein-protein interaction (PPI) analysis showing the top 10 proteins to interact with downregulated proteins. **e** GO analysis (biological process) of upregulated proteins shows an increase in proteins involved in “mitochondria function”

We then assessed protein expression in the SVZ of control (*n* = 4) and PD patients (*n* = 7) (Fig. 4a). Proteome analysis detected 2027 proteins (only proteins that were detected in at least 2 out of 3 donors) of which 90 were differentially expressed in PD in the SVZ homogenate compared to control (Fig. 5a and Additional file 7: Table S7). Panther pathway analysis on downregulated proteins further underlines a dysregulation in pathways involved in Parkinson’s disease (PSMA7, SNCA) as seen in the CD271^+^ NSCs, and also on metabolism (viz. ATP synthesis: CYCS, and glycolysis: GAPDH) (Fig. 5b). GO analysis on downregulated proteins suggest changes in the “protein translocation machinery” (e.g. RPL21, RPL14 and RPS23) and “catabolism of mRNA molecules” (e.g. RPL14 and RPL8) (Fig. 5c). Protein-protein interaction (PPI) analysis showed that proteins involved in “protein folding and transport” (HSPA1 and HSPA8) and “formation of autophagosomes” (MAP1LC3B and MAP1LC3A) were among the top 10 proteins postulated to interact with the downregulated proteins (Fig. 5d). GO term analysis suggest changes in mitochondria function (e.g. UQCRQ, UQCR11 and MUT) (Fig. 5e).

## Discussion

We have previously described a population of NSCs that not only persists in the SVZ at old age, but are also found in the SVZ of PD patients. These cells retain their proliferative and multipotent capacity [52, 54] and are characterized by CD271 expression [55]. The presence of NSCs in PD brains potentially opens up new possibilities for the development of novel therapeutic strategies to stimulate the endogenous NSC pool to replace lost dopaminergic neurons. To further characterize the CD271-expressing NSCs, we performed transcriptome and proteome analysis of cells isolated from the SVZ of both controls and PD patients. Our results provide the gene signature of NSCs from the adult human SVZ and suggest that these CD271^+^ NSCs may transit in a primed-quiescent state, and may thus, be amenable for manipulation to boost their neurogenic potential.

First, we performed in controls a transcriptome profiling of CD271 expressing NSCs by comparing these to SVZ homogenate and CD11b^+^ microglia, which confirmed their NSC identity at transcriptome level and provides a gene signature for NSCs of the adult human SVZ. Our transcriptome and proteome analysis of CD271^+^ NSCs from controls and PD patients revealed several processes and signaling pathways that are altered in PD. Interestingly, Panther pathway analysis showed a dysregulation of the 5-hydroxytryptamine degradation pathway, due to the downregulation of *MAOB* gene expression in PD, which encodes for the enzyme Monoamine Oxidase B that degrades dopamine and serotonin (Fig. 2c). This could reflect an attempt of the system to compensate for the lower level of dopaminergic and serotonergic signaling found in PD. Neurotransmitters have long been known to regulate neurogenesis during development and adulthood [4]. It has been suggested that both dopaminergic and serotonergic signaling promote proliferation in the SVZ [2, 56]. However, a previous study from the lab showed that dopamine had no effect on proliferation of human NSCs in vitro [54]. It is still unclear whether dopamine positively regulates neurogenesis in both NSCs and progenitors. Also, the exact role that neurotransmitter signaling plays in the regulation of neurogenesis *after* injury remains unclear and it might have a different function than under physiological conditions [20, 24].

Our proteomic data suggest a downregulation in glycolytic metabolism (Fig. 4), which has been described as a hallmark of qNSC identity [12, 25, 34, 48]. Moreover, the quiescence marker *FGFR3* was significantly decreased in the SVZ of PD patients. *FGFR3* is enriched in qNSC [40] and is not expressed in proliferating NSCs of the SVZ [15]. Constitutive activation of FGFR3 increases proliferation while decreasing apoptosis in progenitors of the embryonic mouse brain [21]. qNSCs have been shown to downregulate glycolysis and enter a primed-quiescent intermediate state before becoming active and switching to oxidative phosphorylation. Moreover, qNSCs have been shown to enter a primed-quiescent state upon ischemic brain injury in adult mice [34]. In other tissues, such as bone marrow [37] and muscle [43], quiescent stem cells alternate between a quiescent and active state and may enter an “alert state” upon injury, thereby participating to tissue regeneration. Indeed, qNSCs may be more responsive to injury signals, as their higher expression of membrane receptors such as G protein-coupled receptors, may make them more sensitive to changes in their environment [9, 11, 33, 34]. Despite the high variation within groups we took a closer look into genes specifying quiescent or activated NSCs (Fig. 3), lineage specification, and proliferation. Based on these expression profiles we found no evidence for NSC activation or proliferation in PD, thereby confirming earlier results [54]. Thus, in the one hand we found no evidence for increased NSC proliferation, but our proteomic analysis suggest changes in the metabolic and quiescent state of these NSCs. These changes suggest that the CD271^+^ NSCs may transit into an alert state as result of the injury.

Interestingly, we also observed a large number of cilia-related genes that were downregulated in PD (Fig. 2e). These genes are known to be involved in cilia movement, formation, and function. Recently, one of the best known genetic contributors for the development of PD, leucine rich-repeat kinase 2 (LRRK2), has been shown to interfere with primary cilia formation both in vitro and in vivo as a result of its increased kinase activity [10]. Loss of primary cilia on cholinergic neurons led to reduced response to hedgehog signaling, which has been suggested to have neuroprotective effects on dopaminergic neurons [17]. Further studies are necessary to confirm the dysregulation in the expression of cilia-related genes in NSCs of PD patients and its biological significance.

## Conclusions

We provide a first comprehensive overview of the transcriptome and proteome profiles of the human SVZ and NSCs from the human SVZ in health and in PD. Our study confirms the stem cell identity of the CD271^+^ cells and provides the gene signature of NSCs from the adult human SVZ. Our data offers a platform with novel target genes and pathways for manipulation to promote brain repair in PD. This study is the first to show the molecular response of NSCs of the human SVZ to degeneration and provide a first indication that human NSCs of the SVZ may transit into an “alert” state in a neurodegenerative disease. It is important to keep in mind that we investigated the end stage of the disease, and that the transcriptome and proteome profiles offer only a narrow window into the identity of CD271^+^ NSCs. Future studies with single cell RNAseq could circumvent the high variation between donors by providing higher resolution into the profile of this heterogeneous population of NSCs in the adult human brain.

## Additional files

Additional file 1: **Table S1.** Characteristics, diagnosis and pathological evaluation of PD patients and control donors. CNTR = control; PD = Parkinson’s disease; PDD = Parkinson’s disease and dementia; CAA = cerebral amyloid angiopathy; AG = argyrophilic grain; PMD = post-mortem delay; UNK = unknown; ND = not determined; NA = not applicable. (XLSX 13 kb)

Additional file 2: **Table S2.** Characteristics and diagnosis of PD patients and control donors used for immunohistochemical analysis. (DOCX 14 kb)

Additional file 3: **Table S3.** Overview of RNA samples used for RNA sequencing. (DOCX 16 kb)

Additional file 4: **Table S4.** List of qPCR primer sequences. (DOCX 17 kb)

Additional file 5: **Table S5.** Genes differentially expressed in CD271^+^ cells in comparison to SVZ homogenate from control donors and differentially expressed genes in CD271^+^ NSCs from PD patients compared to CD271^+^ cells from control donors. (XLSX 189 kb)

Additional file 6: **Table S6.** Genes differentially expressed in CD271^+^ NSCs in comparison to CD11b^+^ microglia cells from PD and control donors pooled together. (XLSX 120 kb)

Additional file 7: **Table S7.** Proteins differentially expressed in CD271^+^ NSCs and SVZ from PD patients in comparison to control donors. (XLSX 21 kb)

Additional file 8: **Figure S1.** Overview data from donors used for RNAseq analysis. Cntr = control; PD = Parkinson’s disease. *p-value < 0.03; ** p-value < 0.002; *** p-value < 0.0002. Data are presented as mean ± SEM. (TIF 4537 kb)

Additional file 9: **Figure S2.** Overview data from donors used for proteome analysis. Cntr = control; PD = Parkinson’s disease. *** p-value < 0.0002. Data are presented as mean ± SEM. (TIF 4590 kb)

Additional file 10: **Figure S3.** Heatmaps showing expression levels of lineage specific genes for NSC, neuroblasts and oligodendrocytes from controls in CD271^+^ cells and SVZ homogenate. CTRL = control; PD = Parkinson’s disease; NSCs = neural stem cells. (TIF 2029 kb)

Additional file 11: **Figure S4.** Validation of gene expression by qPCR analysis. A select number of genes **(a)** that were identified as differentially expressed in the RNAseq analysis **(b)** were validated. Cntr = control; PD = Parkinson’s disease. *p-value < 0.03, **p-value < 0.002. Data are presented as mean ± SEM. (TIFF 4220 kb)

Additional file 12: **Figure S5.** Differentially expressed proteins involved in metabolism and neurodegenerative diseases. (**a)** Expression level of proteins involved in glycolysis (HK1 and ALDOA), malate metabolism (MDH1) and oxidative phosphorylation (ATP5-). (**b)** Expression level of proteins involved in neurodegenerative diseases. Data shown are average protein expression and TPM-normalized read counts. Cntr = control; PD = Parkinson’s disease. Data are presented as mean ± SEM. (TIF 8689 kb)

## Abbreviations

aNSC: Activated neural stem cells
CTRL: Control
GO: Gene Ontology
NSC: Neural stem cells
PD: Parkinson’s Disease
qNSC: Quiescent neural stem cells
SVZ: Subventricular zone
