# Supplementary material for: Transcriptome and proteome profiling of neural stem cells from the human subventricular zone in Parkinson’s disease
Source: Acta Neuropathol Commun. 2019 Jun 3;7:4. doi: 10.1186/s40478-019-0736-0 (PMC6545684; doi:10.1186/s40478-019-0736-0)
Supplement: Supplementary file 2 — Table S2. Characteristics and diagnosis of PD patients and control donors used for immunohistochemical analysis. (DOCX 14 kb) [file 40478_2019_736_MOESM2_ESM.docx]

**Table S2.** Characteristics and diagnosis of PD patients and control donors used for immunohistochemical analysis.

| **NBB number** | **Sex** | **Age (yrs)** | **Diagnosis** | **PMD (h:min)** | **pH CSF** | **Brain weight** |
| --- | --- | --- | --- | --- | --- | --- |
| 2007-030 | m | 84 | Cntr | 05:35 | 6.98 | 1337 |
| 2007-013 | m | 61 | PD | 07:35 | 6.98 | 1696 |
| 2008-027 | f | 80 | Cntr | 6:55 | 6.50 | 1220 |
| 2008-054 | f | 92 | Cntr | 7:00 | 6.55 | 1230 |
| 2009-005 | m | 82 | Cntr | 05:10 | 6.75 | 1087 |
| 2009-021 | f | 99 | Cntr | 04:15 | 6.63 | 910 |
| 2010-106 | m | 81 | PD | 05:15 | 6.78 | 1416 |
| 2011-079 | m | 81 | PD | 04:30 | 6.42 | 1315 |
| 2011-113 | f | 82 | PD | 03:55 | 6.52 | 1187 |
