# Supplementary material for: Transcriptome and proteome profiling of neural stem cells from the human subventricular zone in Parkinson’s disease
Source: Acta Neuropathol Commun. 2019 Jun 3;7:4. doi: 10.1186/s40478-019-0736-0 (PMC6545684; doi:10.1186/s40478-019-0736-0)
Supplement: Supplementary file 3 — Table S3. Overview of RNA samples used for RNA sequencing. (DOCX 16 kb) [file 40478_2019_736_MOESM3_ESM.docx]

**Table S3** Overview of RNA samples used for RNA sequencing.

| **NBB number** | **Disease** | **Cell type** | **RNA concentration** | **RIN value** | **Total # of reads after mapping** |
| --- | --- | --- | --- | --- | --- |
| 15-018 | Cntr | CD271^+^ NSC | 4.5 ng/µl | 6.6 | 2596700 |
| 15-033 | Cntr | CD271^+^ NSC | 4.6 ng/µl | 8.4 | 2608980 |
| 15-093 | Cntr | CD271^+^ NSC | 6.1 ng/µl | 7.4 | 987648 |
| 18-105 | Cntr | CD271^+^ NSC | 3.1 ng/µl | 8.8 | 4043444 |
| 16-016 | PD | CD271^+^ NSC | 3.9 ng/µl | 8.3 | 1580410 |
| 16-084 | PD | CD271^+^ NSC | 11.6 ng/µl | <6.0 | 3131006 |
| 16-098 | PD | CD271^+^ NSC | 8.6 ng/µl | 8 | 1973143 |
| 16-101 | PD | CD271^+^ NSC | 6.3 ng/µl | 7.6 | 1310307 |
| 16-102 | PD | CD271^+^ NSC | 6.7 ng/µl | <6.0 | 5179727 |
| 16-116 | Cntr | Total SVZ tissue | 21 ng/µl | 6.0 | 715050 |
| 16-137 | Cntr | Total SVZ tissue | 151 ng/µl | 5.4 | 1843488 |
| 17-003 | Cntr | Total SVZ tissue | 79.5 ng/µl | 5.7 | 701138 |
| 17-043 | PD | Total SVZ tissue | 9.9 ng/µl | 6.0 | 1114585 |
| 16-090 | PD | Total SVZ tissue | 21.9 ng/µl | 7.8 | 3227280 |
| 16-098 | PD | Total SVZ tissue | 13.2 ng/µl | 7.2 | 944768 |
| 16-101 | PD | Total SVZ tissue | 25.6 ng/µl | 5.8 | 1847739 |
| 15-087 | Cntr | CD11b^+^ microglia | 43.9 ng/µl | 7.7 | 1415672 |
| 16-116 | Cntr | CD11b^+^ microglia | 15 ng/µl | 7.9 | 2090241 |
| 16-084 | PD | CD11b^+^ microglia | 17.6 ng/µl | 8 | 1170532 |
| 16-090 | PD | CD11b^+^ microglia | 10.1 ng/µl | 7.1 | 1536390 |
| 16-101 | PD | CD11b^+^ microglia | 6.3 ng/µl | 7.7 | 2801397 |
| 16-016 | PD | CD11b^+^ microglia | 10.7 ng/µl | nd | 2544239 |
