# Supplementary material for: Transcriptome and proteome profiling of neural stem cells from the human subventricular zone in Parkinson’s disease
Source: Acta Neuropathol Commun. 2019 Jun 3;7:4. doi: 10.1186/s40478-019-0736-0 (PMC6545684; doi:10.1186/s40478-019-0736-0)
Supplement: Supplementary file 4 — Table S4. List of qPCR primer sequences. (DOCX 17 kb) [file 40478_2019_736_MOESM4_ESM.docx]

**Table S4.** List of qPCR primer sequences.

| **qPCR primers** | | |
| --- | --- | --- |
| **Gene** | **Forward primer (5’-3’)** | **Reverse primer (5’-3’)** |
| *KDM2A* | CAACAGCGATCCCAAGTTAGC | TGGCCGAGTGGGGAATTTAAG |
| *RAD51C* | TTTGGTGAGTTTCCCGCTGTC | AACTTCTTTGCTAAGCTCGGAG |
| *IGFBP5* | TGACCGCAAAGGATTCTACAAG | CGTCAACGTACTCCATGCCT |
| *HSPA1L* | GGCCTTTCCAAGATTGCTGTT | TTCAACATTGCAAACACAGGAAA |
| *E2Ubi* | CTGAAGAGAATCCACAAGGAATTGA | CTCCAACAGGACCTGCTGAAC |
| *ALUS* | CAT GGT GAA ACC CCG TCT CTA | GCC TCA GCC TCC CGA GTA G |
| *EF1α* | AAGCGACCCAAAGGTGGAT | AAGCTGGAAGATGGCCCTAAA |
