# Supplementary figures and images for: Transcriptome and proteome profiling of neural stem cells from the human subventricular zone in Parkinson’s disease
Source: Acta Neuropathol Commun. 2019 Jun 3;7:4. doi: 10.1186/s40478-019-0736-0 (PMC6545684; doi:10.1186/s40478-019-0736-0)

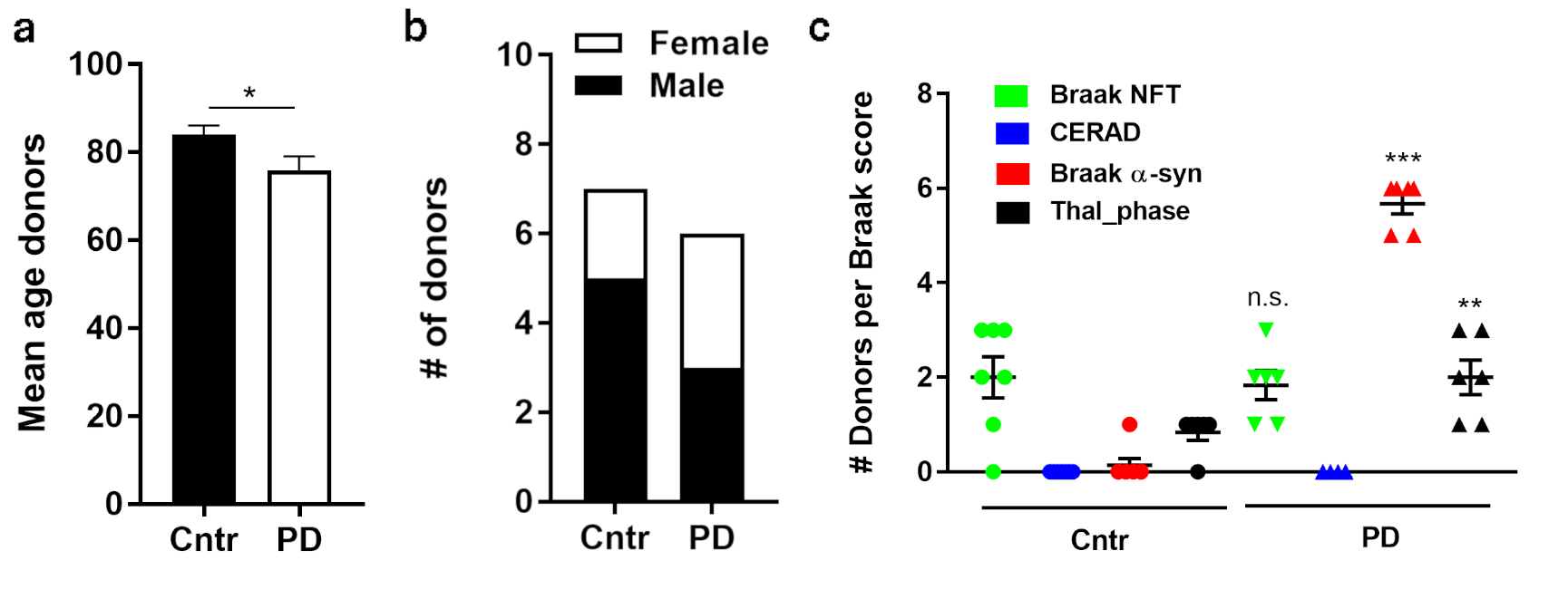

Supplement: Supplementary file 8 — Figure S1. Overview data from donors used for RNAseq analysis. Cntr = control; PD = Parkinson’s disease. *p-value < 0.03; ** p-value < 0.002; *** p-value < 0.0002. Data are presented as mean ± SEM. (TIF 4537 kb) [file 40478_2019_736_MOESM8_ESM.tif]

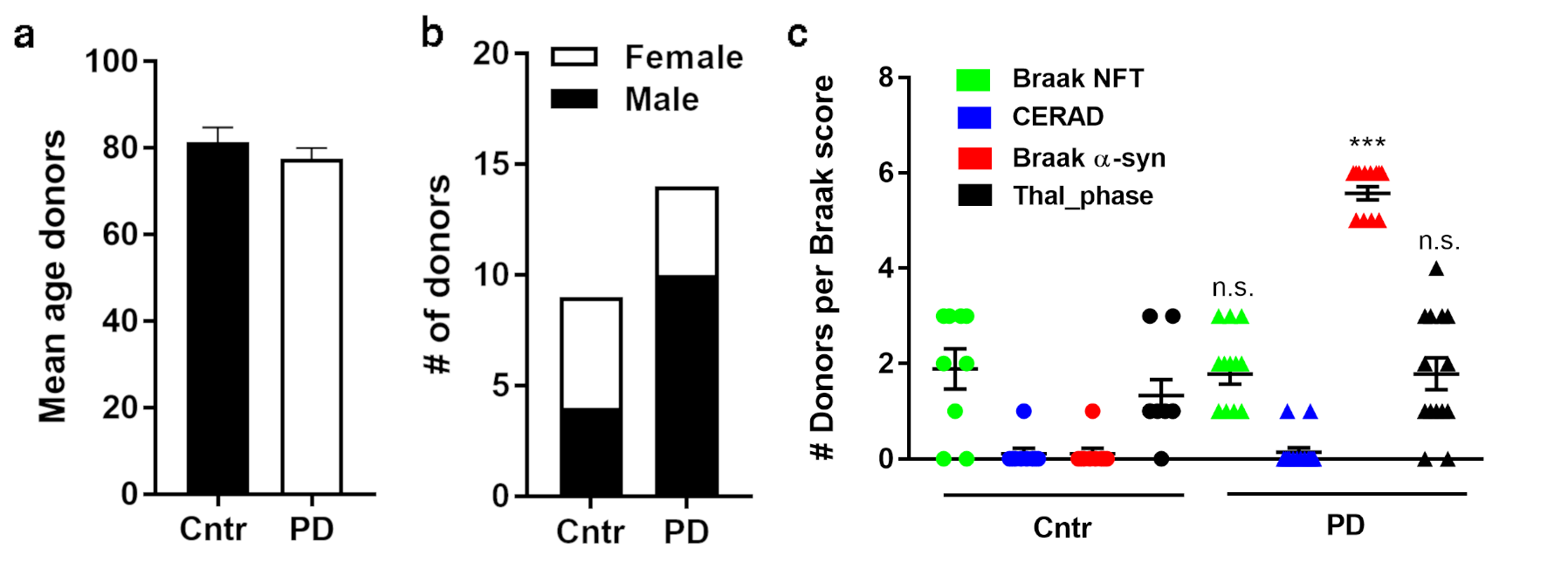

Supplement: Supplementary file 9 — Figure S2. Overview data from donors used for proteome analysis. Cntr = control; PD = Parkinson’s disease. *** p-value < 0.0002. Data are presented as mean ± SEM. (TIF 4590 kb) [file 40478_2019_736_MOESM9_ESM.tif]

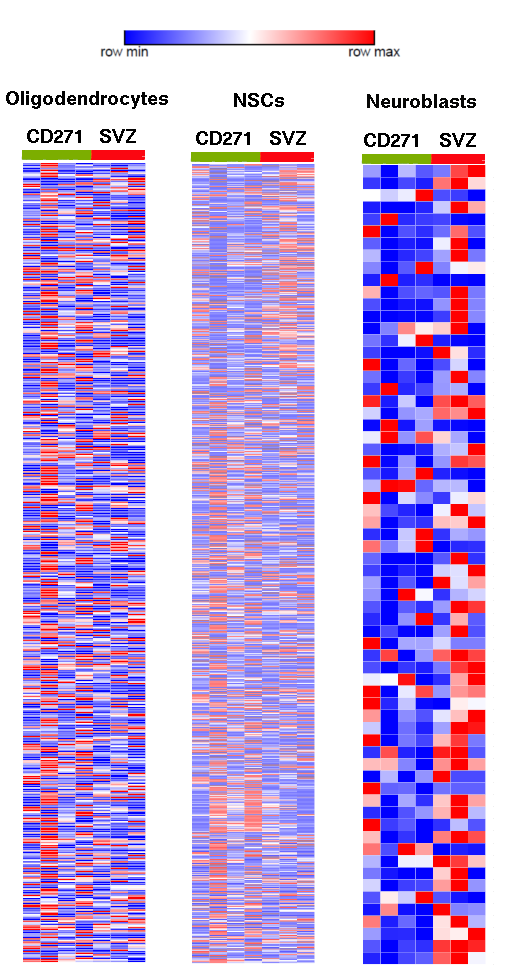

Supplement: Supplementary file 10 — Figure S3. Heatmaps showing expression levels of lineage specific genes for NSC, neuroblasts and oligodendrocytes from controls in CD271+ cells and SVZ homogenate. CTRL = control; PD = Parkinson’s disease; NSCs = neural stem cells. (TIF 2029 kb) [file 40478_2019_736_MOESM10_ESM.tif]

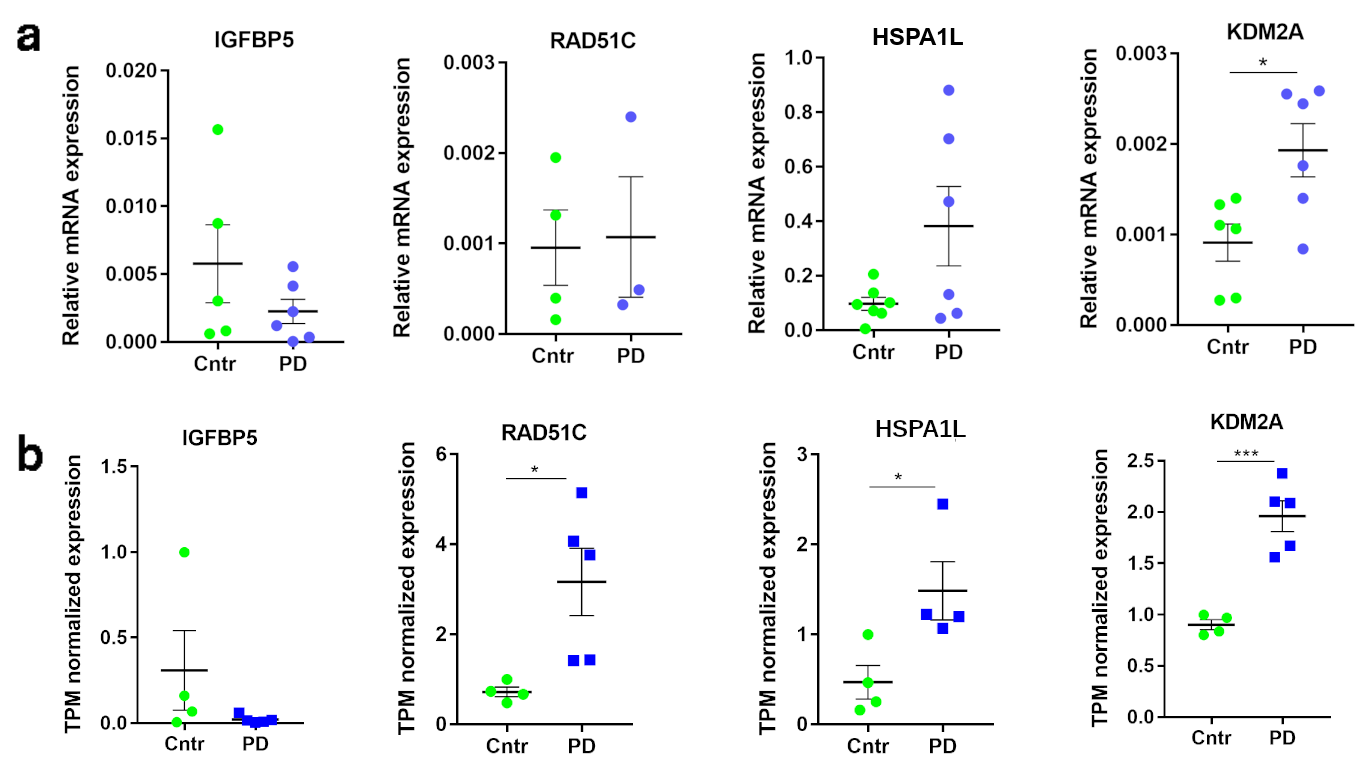

Supplement: Supplementary file 11 — Figure S4. Validation of gene expression by qPCR analysis. A select number of genes (a) that were identified as differentially expressed in the RNAseq analysis (b) were validated. Cntr = control; PD = Parkinson’s disease. *p-value < 0.03, **p-value < 0.002. Data are presented as mean ± SEM. (TIFF 4220 kb) [file 40478_2019_736_MOESM11_ESM.tiff]

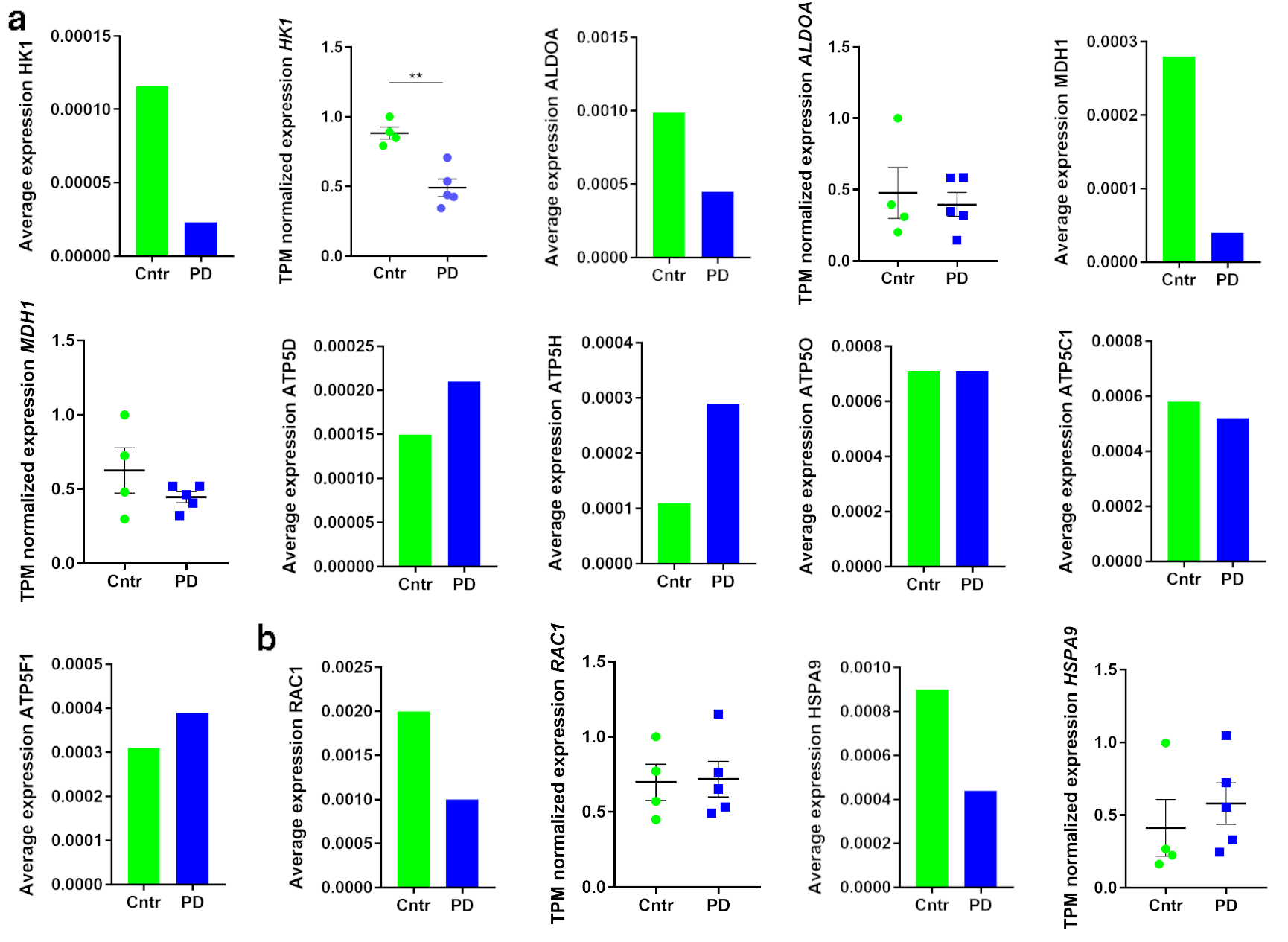

Supplement: Supplementary file 12 — Figure S5. Differentially expressed proteins involved in metabolism and neurodegenerative diseases. (a) Expression level of proteins involved in glycolysis (HK1 and ALDOA), malate metabolism (MDH1) and oxidative phosphorylation (ATP5-). (b) Expression level of proteins involved in neurodegenerative diseases. Data shown are average protein expression and TPM-normalized read counts. Cntr = control; PD = Parkinson’s disease. Data are presented as mean ± SEM. (TIF 8689 kb) [file 40478_2019_736_MOESM12_ESM.tif]
